# Pacinian Neurofibroma of Oral Cavity: A Rare Neurogenic Tumor 

**DOI:** 10.30476/dentjods.2025.104390.2527

**Published:** 2025-12-01

**Authors:** Sara Amanpour, Reza Malekpour Afshar, Alireza Parastar

**Affiliations:** 1 Dept. of Oral and Maxillofacial Pathology, Faculty of Dentistry, Kerman University of Medical Sciences, Kerman, Iran.; 2 Pathology and Stem Cell Research Center, Kerman University of Medical Sciences, Kerman, Iran.; 3 Resident of Oral and Maxillofacial Surgery, Faculty of Dentistry, Kerman University of Medical Sciences, Kerman, Iran.

**Keywords:** Neurofibroma, Pacinian Corpuscles, Oral cavity, Case report

## Abstract

Pacinian neurofibroma is a rare variant of neurofibroma composed of structures resembling pacinian corpuscles at various stages of maturation. It is a rare neurogenic tumor which has been reported predominantly on hands, and feet, where pressure receptors are typically located. It usually presents as a solitary nodule and is not reported in associated with von Recklinghausen’s disease or any other syndrome. The intraoral site is unusual. The purpose of this report is to present a rare case of intra-oral pacinian neurofibroma associated with neurofibromatosis type 1 and to describe its clinical and microscopic features contributing for the best knowledge about this rare entity.

## Introduction

Benign neurogenic tumors are lesions that originate from peripheral nerve bundles and account for about 45% of neoplasms in the head and neck region [ 1 ]. Neurofibroma (NF) is the most common type among these neoplasms [ 2 ] and is described as a benign tumor of peripheral nerve sheath (grade I in WHO classification), composed of diverse lineages cells, including perineurial cells, Schwann cells, fibroblasts with intermixed axons [ 3 ]. NF can occur sporadically or as part of the genetic syndrome neurofibromatosis type 1 (NF1) or Von Recklinghausen disease [ 2 ].

Based upon the clinical presentation and histopathologic features, numerous systems have been made for NF in the literature and also at the European NF meeting (2008) [ 4 ]. One such system classifies NF based on anatomical location into cutaneous NF and subcutaneous deep NF [ 5 ]. Cutaneous NF is classified as diffuse or localized as well. The localized tumors are more common than diffuse lesions, usually presenting as a solitary asymptomatic lesion in adults that can arise in nodular or polypoid form anywhere on the body and it is infrequently related to NF1. However, any patient with multiple lesions should be evaluated clinically for the possibility of NF1. Diffuse cutaneous NF is a rare and clinically unique variant which presents as an ill-defined plaque of thickening in dermal and subcutaneous layers with surface hyperpigmentation. It is most common in young adults and arises on the skin of trunk and also head and neck area. About 10% of the cases are associated with NF1 [ 5 ]. Although unusual microscopic variants of NF1 have been described in the literature, these tumors, in any location, show similar histopathologic and immunohistochemical features. There are 11 different histopathological variants of cutaneous NF based on cell morphology [ 5 ]. Pacinian cutaneous NF is one of these variants, characterized by the presence of numerous components similar to pacinian corpuscles within the classic type of cutaneous NF. Pacinian neurofibroma (PNF) was introduced by Thoma for the first time in 1894 and by Prichard and Custer in 1952 later, and by Prose *et al*. in 1957 as well [ 6 ]. It is usually a solitary nodule that is most commonly developed on the hands, fingers and feet, where many pressure receptors are located [ 7 ]. PNF is not related to von Recklinghausen’s disease or other syndromes [ 8 ].

In the oral cavity, PNF is an uncommon finding and can present as a well-defined, soft, slow-growing mass in the gingival region, which may involve the underlying bone [ 5 ]. The purpose of this study is to report a rare case of intra-oral PNF associated with NF1. 

## Case Presentation

**Figure 1:**
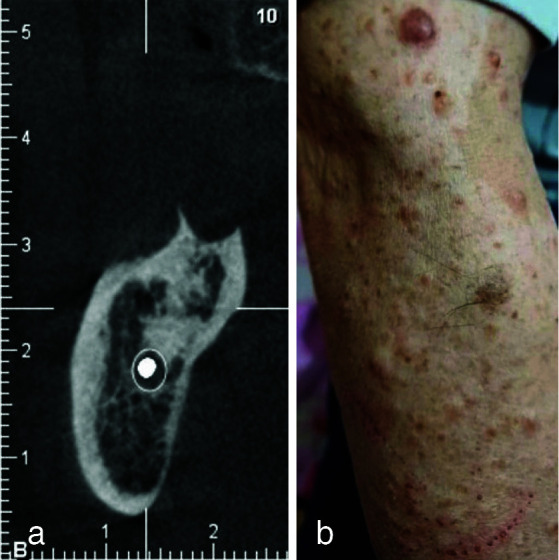
**a:** Cone-beam computed tomography (CBCT) (coronal view) of molar area showing a well-defined opacity. **b:** Skin examination showing multiple papules on the skin of hands

**Figure 2:**
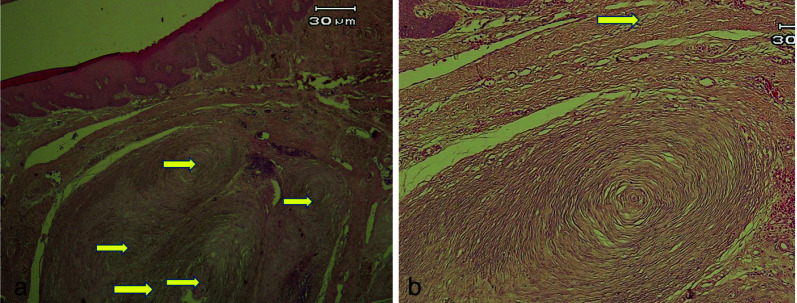
**a:** A well-demarcated tumor with several pacinian corpuscle-like structures (arrows) (Hematoxylin-Eosin, original magnification 40×), **b:** Pacinian corpuscle-like structure: a central homogeneous, hypocellular, eosinophilic core surrounded by pale-staining, concentric collagenous lamellae (arrows). (Hematoxylin-Eosin, original magnification 100×)

A 48-year-old man was referred to a dental clinic in Kerman in 2024, complaining of a painless gingival mass that interfered with the placement of his partial denture. Oral examination revealed a dome-shaped mass on the lingual attached gingiva of the right mandibular ridge in front of the retromolar pad area. The mass had the same color as the surrounding normal mucosa, was covered by intact mucosa, and measured about 2 cm in diameter. It was sessile, and had firm consistency upon palpation According to the patient; the lesion had been present for about a year with slow growth and was not associated with trauma or other factors. No enlarged cervical lymph nodes were observed. A cone-beam computed tomography (CBCT) scan revealed a small, well-defined, elliptical radiopacity with homogeneous density without a surrounding radiolucent rim, suggestive of osteosclerosis in the molar region ("Figure 1a). However, no abnormalities were detected in relation to the soft tissue lesion. Skin examination revealed multiple papules and nodules on the face, trunk, and extremities, with higher nodule density on the face and hands (Figure 1b). The patient reported that he had been diagnosed with NF1 for 30 years. Based on the clinical examination and patient history, a differential diagnosis of an intra-oral NF was considered. The lesion was surgically excised, and the specimen was sent to a pathology laboratory. Histopathological examination, using hematoxylin and eosin staining (H&amp;E; X 40 &amp; 100), revealed a well-demarcated tumor with nerve bundles and several pacinian corpuscle-like structures, each showing a central homogeneous, hypocellular, eosinophilic core surrounded by pale-staining, concentric collagenous lamellae with a few nerve bundles in a collagenous stroma. The lobules contained ovoid or spindle-shaped nuclei, both in the central portion and surrounding lamellae. The concentric lamellae were merging with adjacent collagen fibers, suggestive of PNF (Figures 2a-b). 

**Figure 3:**
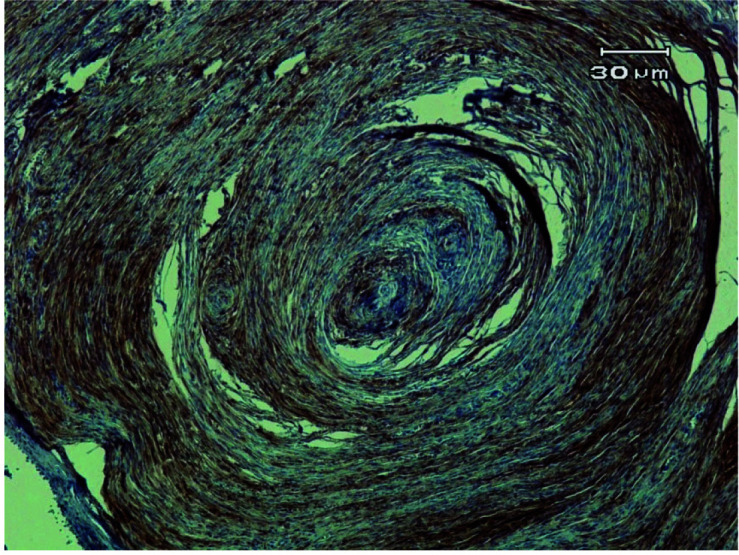
Immunohistochemistry (IHC) staining for protein S-100 in pacinian neurofibroma (100×)

Considering this histopathologic picture, the diagnosis of PNF was made. Although the lesion was identifiable using routine hematoxylin and eosin staining, the specimen was submitted for immunohistochemistry for protein S-100, which showed positive reactivity to this neural cell marker (Figure 3). 

Sutures were removed one week later, and the patient was on regular follow-up with no complaints or recurrence six months after surgery. 

## Discussion

Vater-Pacinian corpuscles are pressure-sensitive, oval-shaped mechanoreceptors located at the ends of sensory nerve fibers, primarily in the palmar and plantar surfaces of the hands and feet. Other sites include the conjunctiva, vulva, clitoris, buttocks, urethra and loose connective tissue. These structures are tactile receptors [ 5 ]. PNF is an uncommon variant of NF composed of structures similar to pacinian corpuscles at various stages of maturation. The mature structures show a homogeneous, acellular and eosinophilic central core, while immature corpuscles contain more cellular elements with spindle nuclei [ 4 ]. PNF is a rare benign neural tumor, typically presenting as a solitary, soft-to-firm, well-demarcated and mobile nodule, as in our case. Multiple tumors are uncommon. Generally, they occur on the hands and feet, although other locations include the buttocks, sacrococcygeal region, arm, neck and face. They are more frequently seen in adolescents and young adults [ 5 ]. 

Given the lesion's location on the alveolar ridge adjacent to a partial denture, the clinical differential diagnosis included reactive lesions such as peripheral ossifying fibroma, pyogenic granuloma, and giant cell granuloma as well as other benign soft tissue tumors. 

The fact that many PNF present as common oral mucosal pathologies highlights the critical importance of conducting histopathological examinations on all excised lesions, regardless of its benign clinical appearance, to ensure an accurate diagnosis.

Histopathologic examination plays an important role in verifying the diagnosis, as clinical features are not typical enough in most cases [ 1 ]. In this case, microscopic characteristics included a well-defined lesion with round or ovoid lobules containing pacinian corpuscles in a fibrous stroma. Immunohistochemistry showed tumor cells with positive immunoreactivity for S-100 antibodies.

Differential diagnoses included pacinian hypertr-ophy, pacinian hyperplasia, and NF. Pacinian hyperplasia is a hamartomatous overgrowth and there is usually a history of prior trauma. In both pacinian hypertrophy and pacinian hyperplasia, the classical structure of pacinian corpuscles is well-remained. In contrast, PNF shows pacinian corpuscle-like differentiation at various stages of maturation within a myxoid or fibrous stroma [ 8 ]. Other histopathologic differential diagnoses include ancient schwannoma and peripheral nerve sheath myxoma. Although both tumors originate from neural tissue and are S-100 positive, their microscopic features play the main role in differentiating between them. Ancient schwannoma is characterized by Antoni A and B areas, along with degenerative changes such as stromal myxoid degeneration, cystic changes, hemorrhage, and hemosiderin pigmentation. In contrast, peripheral nerve sheath myxoma exhibits a lobulated prominent myxoid stroma containing numerous spindle and stellate cells . The presence of pacinian corpuscle-like structures and a collagenous stroma in PNF serves as a diagnostic hallmark.

Based on the literature, PNF is not associated with von Recklinghausen’s disease or other syndromes that usually needs further workup [ 8 ]. This is the first case of PNF associated with NF1. The clinical course of this tumor is benign; however, recurrence rate is 50% [ 5 ]. Different clinical presentations can make the diagnosis difficult, leaving the definite diagnosis to the pathologist [ 1 ]. Wide surgical excision is the treatment of choice, considering crucial factors such as tumor size and invasion to the vascular and nerve structures [ 8 ].

The high percentage of neural tumors in the head and neck region (45%) is attributed to the large number of peripheral nerve endings in this area. The rare lesions reported in the oral cavity may originate from the nerve endings of the inferior alveolar nerve, triggered by trauma or an unknown stimulus [ 1 ]. In our case, the patient’s history of NF1 suggests a higher likelihood of NF occurrence in the mouth. This case was reported with the informed consent of the patient. 

## Conclusion

In the literature, there are few reports of PNF, which is typically found on the hands, feet, buttocks, and rarely in the oral cavity. To our knowledge, this is the first case of PNF associated with NF1 presented in the oral cavity. Histopathological analysis is crucial for diagnosis, particularly the identification of structures resembling Vater-Pacini corpuscles.

## References

[1] Souza LB, Oliveira JMB, Freitas TMC, Carvalho RA ( 2003). Pacinian neurofibroma: report of a rare intraoral case. Braz J Otorhinolaryngol.

[2] Neville B, Damm DD, Allen CM, Chi AC (2016). Oral and maxillofacial pathology.

[3] Sbaraglia M, Bellan E, Dei Tos AP ( 2021). The 2020 WHO classification of soft tissue tumours: news and perspectives. Pathologica.

[4] Ortonne N, Wolkenstein P, Blakeley JO, Korf B, Plotkin SR, Riccardi VM, et al ( 2018). Cutaneous neurofibromas: Current clinical and pathologic issues. Neurology.

[5] Nagrani NS, Bhawan J ( 2023). Histopathological variants of cutaneous neurofibroma: a compendious review. Dermatopathology (Basel).

[6] Nath AK, Timshina DK, Thappa DM, Basu D ( 2011). Pacinian neurofibroma: a rare neurogenic tumor. Indian J Dermatol Venereol Leprol.

[7] Deshpande GU, Bhatoe HS, Ramji R, Panicker NK ( 1997). Pacinian neurofibroma of the scalp. Med J Armed Forces India.

[8] Panhotra SH, Jairajpuri ZS, Sultan B, Jetley S, Ahmad N ( 2020). Rare Neurogenic Tumor- Pacinian Neurofibroma. Ann Pathol Lab Med.

[9] Bajpai M, Pardhe N ( 2017). Ancient schwannoma of gingiva: A rare case report. J Indian Soc Periodontol.

[10] Frydrych AM, Firth NA ( 2018). Oral nerve sheath myxoma: a rare and unusual intraoral neoplasm. Clinical Case Reports.

